# Vascular endothelial growth factor-A (VEGF-A) and chemokine ligand-2 (CCL2) in Amyotrophic Lateral Sclerosis (ALS) patients

**DOI:** 10.1186/1742-2094-8-47

**Published:** 2011-05-13

**Authors:** Pawan K Gupta, Sudesh Prabhakar, Suresh Sharma, Akshay Anand

**Affiliations:** 1 Department of Neurology, Post Graduate Institute of Medical Education and Research (PGIMER), Chandigarh-160012, India; 2 Department of Statistics, Panjab University, Chandigarh-160012, India

## Abstract

**Background:**

Vascular endothelial growth factor-A (VEGF-A) and chemokne ligand-2 (CCL2) levels have been examined in Amyotrophic Lateral Sclerosis (ALS) patients in Western countries. We measured these values in North Indian ALS patients, since these patients display considerably enhanced survival duration.

**Methods:**

Sporadic ALS patients were included on the basis of El Escorial criteria. VEGF-A and CCL2 levels were analyzed in serum and cerebrospinal fluid (CSF) of 50 ALS patients using enzyme linked immunosorbent assay (ELISA) and compared with normal controls. Their levels were adjusted for possible confounders like cigarette smoking, alcohol and meat consumption.

**Results:**

Contrary to previous studies, VEGF-A was found to be elevated significantly in serum and CSF in ALS patient population studied. We also found an increase in CCL2 levels in CSF of these ALS patients. Serum and CSF from definite ALS revealed higher VEGF-A as compared to probable and possible ALS. CCL2 was unaltered between definite, probable and possible ALS. Univariate and multivariate analysis revealed a lack of association of smoking, alcohol and meat consumption with VEGF-A and CCL2 levels.

**Conclusions:**

VEGF-A upregulation may indicate an activation of compensatory responses in ALS which may reflect or in fact account for increased survival of North Indian ALS patients after disease onset. The intrathecal synthesis of CCL2 suggests the involvement of adult neural stem cells and microglial activation in ALS pathogenesis which needs further investigation.

## Introduction

Amyotrophic lateral sclerosis (ALS) is a neurodegenerative disorder with genetic and clinical heterogeneity. Existing evidence suggests that Vascular endothelial growth factor-A (VEGF-A) delivery delays the onset and progression of ALS in superoxide dismutase-1 (SOD1) mutated transgenic mouse model by activating PI3-K/Akt anti apoptotic pathway [1,2]. VEGF-A also shown to be involved in proliferation and differentiation of adult mouse neural progenitor's cells [3]. On the other hand, chemokine ligand-2 (CCL2), a proinflammatory molecule, enhances microglial recruitment after injury to central nervous system (CNS) and exacerbates ALS [4]. It has been observed that CCL2 knockout mice have reduced involvement of immune cells and are resistant to stroke and autoimmune encephalomyelitis [5,6]. Reports suggest that VEGF-A_165_, a major isoform of VEGF-A, and CCL2 may interact in synergistic manner to mount a response to disease [7]. We therefore hypothesized that VEGF-A-CCL2 axis plays a crucial role in ALS pathogenesis and could be a target for development of future therapy for ALS.

## Subjects and methods

50 patients diagnosed with ALS were recruited after obtaining informed consent as per institute ethical committee guidelines (No. 7055-PG-1Tg-05/4348-50). All patients were born in Northern India. Of the patients examined, 25 patients fulfilled the "El Escorial criteria" for definite ALS, 15 individuals fulfilled for probable and 10 patients as possible ALS at the time of sample collection. Based on ALS-Functional Rating Score [8], 11 patients had respiratory insufficiencies alongwith orthopnea and dyspnea, although none of the patients needed respiratory support. There were 42 cases of limb and 8 cases of bulbar onset ALS. Patients with history of stroke, pre-eclampsia, diabetic neuropathy, glaucoma, diabetes, those who have been receiving riluzole, anti inflammatory drugs, antioxidants or other treatment were excluded. 50 genetically unrelated healthy normal controls without any complaints of hypertension, diabetes, heart disease etc were also included. Cerebrospinal fluid (CSF) from 42 subjects without any CNS disorders but undergoing routine spinal anesthesia for surgery was collected and considered control sample. Subjects were categorized as cigarette smokers and never smokers, alcohol consumers and nonalcoholics, vegetarian and non-vegetarian (or meat consumers) through a questionnaire [9]. The characteristics of subjects have been reproduced in Table 1.

**Table 1:** Characteristics of subjects

Subjects	Age (y)^†^	M/F (n)	Age of onset (y)	Disease duration^‡ ^(mo)	Smokers (n)	Alcohol consumers (n)	Non-vegetarian (n)	Total protein (g/l)^†^	
								CSF	Serum
ALS	47.4 ± 12.4	38/12	46.2 ± 12.8	19.0 ± 12.7	12	12	20	0.43 ± 0.2	48.2 ± 26.7

Controls (Serum)	40.0 ± 12.8	39/11			10	14	27		48.7 ± 28.7

Controls (CSF)	43.4 ± 17.1	35/07			08	09	10	0.42 ± 0.1	

Clinical and demographic summary of ALS cases and controls. n, Number; M, male; F, female; y, years; mo, months; g, gram; l, litre; CSF, cerebrospinal fluid; Age, age of onset, duration of disease, CSF and serum total protein are indicated as mean ± standard deviation (SD). † One way ANOVA followed by LSD post hoc analysis showed that mean age and, mean concentration of total protein in serum and CSF did not differ significantly among the groups (p > 0.05). ‡ Duration of disease is the interval between appearance of first symptom of ALS and collection of sample. ALS subjects were asked to provide all clinical and demographic details at the age of disease-onset.

Serum was separated from 4.0 ml blood collected in serum separator tube (BD Biosciences, USA). ~2.0 ml CSF was drawn in a sterilized container. Serum and CSF was stored at -80°C until assayed.

Serum VEGF-A, CCL2 and CSF CCL2 was measured using Quantikine sandwich enzyme linked immunosorbent assays (ELISA; R&D systems, USA) and read at 450 nm in 680XR microplate reader (Biorad, USA). CSF VEGF-A levels were quantitated with QuantiGlo chemiluminescent assay (R&D systems), and read as relative light units in luminometer (Biotek, USA). ELISA kits for VEGF-A measured unbound natural human VEGF-A_165 _splice variant.

Mann Whitney U test and one-way analysis of variance (ANOVA) followed by Fisher's least significant difference (LSD) post hoc analysis was applied to analyze skewed and normally distributed data respectively. As smoking, alcohol and meat consumption may affect VEGF-A and CCL2 levels, crude odds ratio (OR) of their association was evaluated by univariate logistic regression. Adjusted OR to investigate independent effect of these covariates was computed using multivariate logistic regression and χ^2 ^(chi square) test was performed to calculate *p*-value. *p*-value was considered significant at ≤ 0.05. Statistical analysis was performed by statistical package and service solutions 16.

## Results

**Figure 1:**
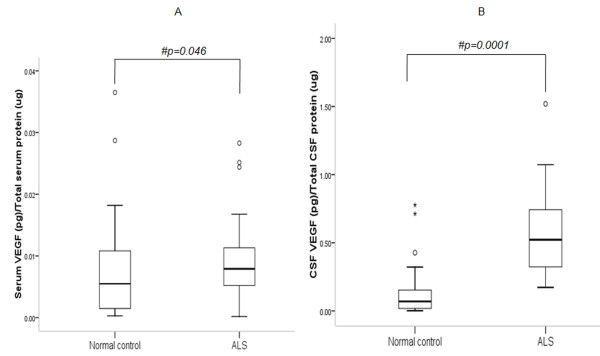
**Level of VEGF-A in serum (A) and CSF (B) of ALS patients and normal controls**. Boxes include values from first quartile (25th percentile) to third quartile (75th percentile). Lower and upper error bar refers to 10th and 90th percentile respectively. The thick horizontal line in the box represents median for each dataset. Levels of VEGF-A were normalized to total serum and CSF protein respectively. Outliers and extreme values are shown in circles and asterisk respectively. # indicates significant difference (p < 0.05) between the given conditions. Data was analyzed by Mann Whitney U Test. ALS, Amyotrophic Lateral Sclerosis; VEGF-A, vascular endothelial growth factor-A; pg, picogram; μg, microgram.

**Figure 2:**
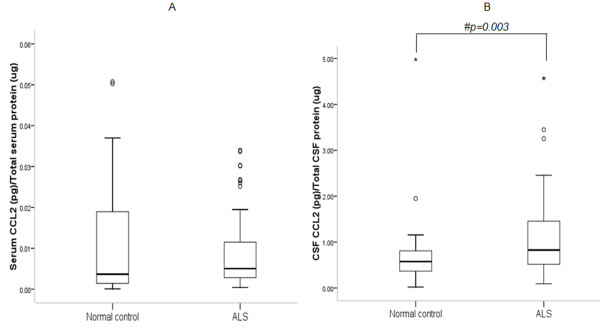
**Level of CCL2 in serum (A) and CSF (B) of ALS patients and normal subjects**. Boxes include values from first quartile (25th percentile) to third quartile (75th percentile). Lower and upper error bar refers to 10th and 90th percentile respectively. The thick horizontal line in the box represents median for each dataset. Levels of CCL2 were normalized to total protein in the serum and CSF samples. Outliers and extreme values are shown in circles and asterisk respectively. # indicates significant difference (p < 0.05) between the given conditions. Data was analyzed by Mann Whitney U Test. ALS, Amyotrophic Lateral Sclerosis; CCL2, chemokine ligand 2; pg, picogram; μg, microgram.

**Figure 3:**
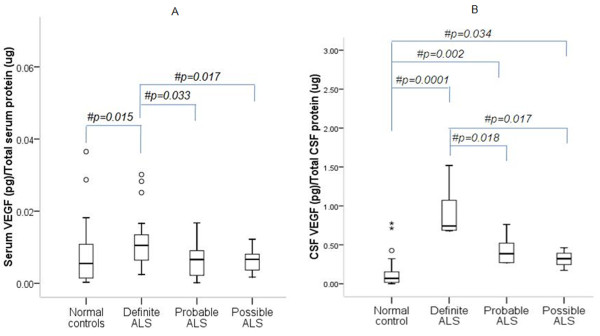
**Level of VEGF-A in serum (A) and CSF (B) of definite ALS, probable ALS, possible ALS patients and normal subjects**. Boxes include values from first quartile (25th percentile) to third quartile (75th percentile). Lower and upper error bar refers to 10th and 90th percentile respectively. The thick horizontal line in the box represents median for each dataset. Levels of VEGF-A were normalized to total protein in the serum and CSF samples. Outliers and extreme values are shown in circles and asterisk respectively. # indicates significant difference (p < 0.05) between the given conditions. Data was analyzed by Mann Whitney U Test. ALS, Amyotrophic Lateral Sclerosis; VEGF-A, vascular endothelial growth factor-A; pg, picogram; μg, microgram.

**Figure 4:**
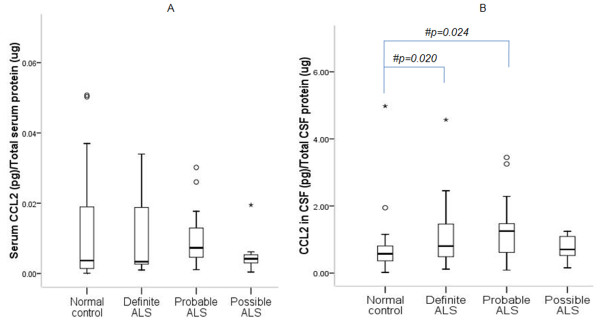
**Level of CCL2 in serum (A) and CSF (B) of definite ALS, probable ALS, possible ALS patients and normal subjects**. Boxes include values from first quartile (25th percentile) to third quartile (75th percentile). Lower and upper error bar refers to 10th and 90th percentile respectively. The thick horizontal line in the box represents median for each dataset. Levels of CCL2 were normalized to total protein in the serum and CSF samples. Outliers and extreme values are shown in circles and asterisk respectively. No significant difference (p > 0.05) was found between the given conditions. Data was analyzed by Mann Whitney U Test. ALS, Amyotrophic Lateral Sclerosis; CCL2, chemokine ligand 2; pg, picogram; μg, microgram.

ELISA indicates elevated serum VEGF-A in ALS as compared to controls (Figure 1A; p = 0.046). Median CSF VEGF-A concentration was significantly higher in ALS patients than controls (Figure 1B; p = 0.0001). No difference in serum CCL2 was observed between ALS and controls (Figure 2A; p > 0.05). However, CSF CCL2 was found increased in ALS as compared to controls (Figure 2B; p = 0.003). There was elevated serum VEGF-A in definite ALS in comparison to controls, probable and possible ALS (Figure 3A; p = 0.015, p = 0.033 and p = 0.017 respectively). Similarly, CSF from definite ALS was reported to have statistically higher VEGF-A than controls, probable and possible ALS (Figure 3B; p = 0.0001, p = 0.018 and p = 0.017 respectively). However, serum and CSF CCL2 levels did not differ among definite, probable and possible ALS (Figure 4A-4B; p > 0.05).

No association of cigarette smoking, alcohol and meat consumption with VEGF-A (Table 2) and CCL2 (data not shown) levels in serum and CSF was observed upon univariate and multivariate analysis.

**Table 2:** Crude and adjusted OR for VEGF-A levels in smokers, alcohol and meat consumers

	OR (95% CI)^†^	p*	Adj. OR (95% CI) ^‡^	p*
**Serum VEGF-A**				
Smoking	1.4 (0.4-4.5)	0.5	0.7 (0.2-2.5)	0.6
Alcohol consumption	0.7 (0.2-2.2)	0.6	0.9 (0.3-3.0)	0.9
Meat consumption	1.5 (0.5-3.9)	0.4	1.4 (0.5-4.0)	0.7

**CSF VEGF-A**				
Smoking	1.4 (0.4-4.5)	0.4	0.7 (0.2-2.5)	0.6
Alcohol consumption	0.7 (0.2-2.2)	0.4	0.9 (0.3-3.0)	0.8
Meat consumption	1.5 (0.5-3.9)	0.2	1.4 (0.5-4.0)	0.2

Never smoking/Nonalcoholic/Vegetarian**	1.0		1.0	

† Univariate logistic regression was used to evaluate crude OR. ‡ Multivariate logistic regression has been used to adjust the effect of smoking on VEGF-A levels with alcohol and meat consumption as covariates. Likewise, effect of alcohol is adjusted for smoking and meat consumption, and meat consumption is corrected for smoking and alcohol intake. * χ^2 ^(chi square test) was used to test the level of significance. ** Never smoking, nonalcoholic and vegetarian diet is considered as reference group. OR, odds ratio; CI, confidence interval; Adj, adjusted.

## Discussion

Our study of North Indian ALS patients represents a unique opportunity to understand the disease from a distinct genetic perspective, given that these patients bring a peculiar spectrum of extended life expectancy in comparison to Western counterparts [10]. An earlier Indian study has reported the median survival duration of 114.83 ± 25.9(SE) months in 1153 ALS patients after disease onset. The mean survival duration of male patients was 110 ± 27.4(SE) months which was not significantly different from mean survival duration of female [118.9 ± 6.3(SE) months] patients [11], while, patients from USA and Europe make up 3-6 years of survival time [12,13].

The increased serum VEGF-A in our ALS patients is consistent with the existing report [14], and suggests that serum is a pathophysiologically relevant fluid in ALS, particularly in definite ALS, however, a few other studies have failed to observe significant difference in plasma and serum VEGF-A levels in ALS patients [15,16].

The elevated CSF VEGF-A in ALS indicates possible presymptomatic initiation of pathological events and intrathecal production from degenerating motor neurons, which is contrary to existing study where decreased VEGF-A_165 _dependent neuroprotection in ALS, due to reduced CSF VEGF-A, has been suggested [15]. Since the impaired VEGF-A expression in early ALS negatively influences the clinical outcome of the disease [15], its upregulation suggests a compensatory response, contributing to prolonged survival of our ALS patients. It is speculated that North Indian ALS patients generate increased VEGF-A or stimulate glutamate receptor-2 expression to ameliorate excitotoxicity [17,18]. We are unable to conclude whether this response is a consequence of amyotrophy, hypoxia, dietary or other environmental factors, or some subtle genetic differences [14,15].

**Figure 5:**
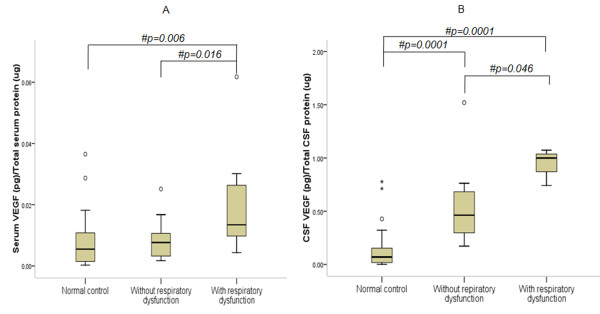
**Level of VEGF-A in serum (A) and CSF (B) of ALS patients with respiratory dysfunction**. Boxes include values from first quartile (25th percentile) to third quartile (75th percentile). Lower and upper error bar refers to 10th and 90th percentile respectively. The thick horizontal line in the box represents median for each dataset. Levels of VEGF-A were normalized to total serum and CSF protein respectively. Outliers are shown in circles. # indicates significant difference (p < 0.05) between the given conditions. Data was analyzed by Mann Whitney U Test. ALS, Amyotrophic Lateral Sclerosis; VEGF-A, vascular endothelial growth factor-A; pg, picogram; μg, microgram.

Further analysis shows significantly increased VEGF-A in 11 ALS patients with respiratory dysfunction indicating a possible association with hypoxia (Figure 5A-5B; Table 3). Elevated VEGF-A in serum and CSF of definite ALS suggests extensive involvement of neuroaxis and relatively higher degree of neurodegeneration and regeneration than probable and possible variants. No significant difference in median disease duration between definite [14(4-36) months], probable [16(3-72) months] and possible ALS [14(9-24) months] cases was observed. It must be pointed out that while the disease duration was accurately documented, the actual survival time of patients after disease onset could not be ascertained.

**Table 3:** Clinical summery of 11 ALS patients with respiratory dysfunction

ALS subjects	El Escorial criteria	ALSFRS-R	Impairment	Disease duration at sample collection (mo)^‡^
Patient 1	Definite	27	Moderate	12

Patient 2	Definite	23	Severe	12

Patient 3	Definite	18	Severe	24

Patient 4	Definite	16	Severe	18

Patient 5	Definite	29	Moderate	12

Patient 6	Definite	29	Moderate	04

Patient 7	Definite	29	Moderate	09

Patient 8	Definite	31	Moderate	08

Patient 9	Definite	35	Moderate	24

Patient 10	Probable	34	Moderate	12

Patient 11	Probable	35	Moderate	30

ALSFRS-R: ALS functional rating score-revised; Impairment was measured with ALSFRS-R. ‡ Duration of disease is the interval between appearance of first symptom of ALS and collection of sample. Median disease duration in patients with respiratory dysfunction is 12(4-30) months whereas median duration of disease was 17(3-72) months in patients without respiratory dysfunction, although the observed difference was not significant upon Mann-Whitney U analysis (p > 0.05).

Intrathecal secretion of CCL2 may offer neuroprotection against glutamate excitotoxicity either by reducing N-Methyl-D-aspartate (NMDA)-dependant release of glutamate and/or increasing astrocytes efficiency to clear synaptic cleft glutamate [19]. CCL2 promotes CCR2 and CCR5 expressing C17.2 neural progenitor cell migration and their differentiation into neuronal and glial phenotype [20,21]. CCL2 is also known to be angiogenic and participates in hypoxia inducible factor-1α induced VEGF-A expression [7]. Likewise, VEGF-A upregulates CCL2 expression by activating nuclear factor-kB via extracellular signal-regulated kinases (ERK) pathways in microglia [22]. Hence, it is possible that VEGF-A-CCL2 axis plays a crucial role in ALS pathogenesis.

The comparison between normal and neurological controls showed relatively pronounced increase in VEGF-A and CCL2 in the latter thus limiting the utility of these as biomarkers in ALS, however, further analysis of this data is being conducted by including homogeneous Parkinson's disease controls instead of the heterogeneous neurological controls used (data not shown).

Regardless of the reason, it is clear that elevated VEGF-A is associated with prolonged survival of Indian ALS patients. At this time, we have no explanation for the basis of this finding, but discuss its therapeutic potential. Increasing brain levels of VEGF-A by genetic engineering, direct infusion or stem cell transplantation may provide limited but significant prolonged life expectancy of afflicted patients. Animal and culture studies have documented that aside from promoting revascularization, VEGF-A is a potent vasodilator, as well as attractant of marrow stromal cells and to some extent, hematopoietic stem cells [23]. Our results suggest that etiological factor VEGF-A needs to be augmented alongwith regulation of inflammation, as noted here by consistently increased CCL2, either by hematopoietic cell transplantation or by defining other neurotrophic and proinflammatory factors involved in ALS. Localized stem cell therapy designed to restore lost neural tissue presents subsequent therapeutic interventions [24]. Such a multifaceted approach will be helpful in presymptomatic identification of individuals who will develop ALS. These individual thus can be treated to prevent ALS from rapidly overwhelming the host defenses.

The lack of association between smoking, alcohol and meat consumption with VEGF-A and CCL2 enhances the credibility of the results since these confounders increased the endpoints assessed but did so regardless of ALS status of the individual studied. The current report thus reliably indicates that ALS is associated with increased VEGF-A and CCL2 providing a foundation for subsequent studies to examine if this result is a factor in the enhanced survival of Indian ALS patients and if so, the mechanisms involved.

## Conclusions

Our study supports that VEGF-A and CCL2 may be involved in enhancing the survival time of sporadic ALS patients, however, comprehensive understanding of growth factors network is required to unveil diagnostic and therapeutic efficacy of these molecules.

## Abbreviations

ALS: amyotrophic lateral sclerosis; ANOVA: analysis of variance; CCL2: chemokine ligand-1; CCR2: chemokine receptor-2; CSF: cerebrospinal fluid; ELISA: enzyme liked immunosorbent assay; ERK: extracellular signal-regulated kinases; LSD: least significant difference; NMDA: N-Methyl-D-aspartate; SE: standard error; SOD1: superoxide dismutase 1; PI3-K: phosphatidylinositol 3-kinases; VEGF: vascular endothelial growth factor.

## Ethical approval

Ethical approval was obtained by institute ethical committee, PGIMER, Chandigarh, India - 160012 (No. 7055-PG-1Tg-05/4348-50).

## Competing interests

The authors declare that they have no competing interests.

## Authors' contributions

PKG Acquisition of data and writing of manuscript; SP inclusion of patients, grant PI and clinical scoring; SS Statistical analysis; AA Interpretation and analysis of data, grant co PI and editing of manuscript. All authors read and approved the final manuscript.
