# Vascular endothelial growth factor-A (VEGF-A) and chemokine ligand-2 (CCL2) in amyotrophic lateral sclerosis (ALS) patients

**DOI:** 10.1186/1742-2094-8-97

**Published:** 2011-08-11

**Authors:** Pawan K Gupta, Sudesh Prabhakar, Suresh Sharma, Akshay Anand

**Affiliations:** 1 Department of Neurology, Post Graduate Institute of Medical Education and Research (PGIMER), Chandigarh-160012, India; 2 Department of Statistics, Panjab University, Chandigarh-160012, India

## Abstract

Correction to Gupta P K, Prabhakar S, Sharma S, Anand A. Vascular endothelial growth factor-A (VEGF-A) and chemokine ligand-2 (CCL2) in amyotrophic lateral sclerosis (ALS) patients. *Journal of Neuroinflammation *8:47.

## Correction

The authors observe that, in Table two of our study [1], crude ORs and adjusted ORs for serum VEGFA were also repeated for CSF VEGFA, however, these values for CSF VEGFA are different from serum VEGFA. Correct OR values of CSF VEGFA are given below.

Smoking: [crude OR(95% CI) = 1.7 (0.3-9.1), p = 0.4; adj OR (95% CI) = 0.6 (0.08-4.4), p = 0.6].

Alcohol consumption: [crude OR(95% CI) = 0.6 (0.1-2.2), p = 0.4; adj OR (95% CI) = 0.8 (0.1-4.4), p = 0.8].

Meat consumption: [crude OR(95% CI) = 2.2 (0.6-7.7), p = 0.2; adj OR (95% CI) = 2.1 (0.5-7.7), p = 0.2].
